# Multidimensional responses to generative AI in higher education: an integrative framework linking institutional governance, teaching practice, and student psychological adaptation

**DOI:** 10.3389/fpsyg.2026.1789506

**Published:** 2026-05-05

**Authors:** Weihua Kuang, Xiaoqing Gao

**Affiliations:** 1 School of Education Science, Hunan Normal University, Hunan, Changsha, China; 2 School of International Business, Hunan University of Information Technology, Hunan, Changsha, China

**Keywords:** educational governance, generative AI, higher education governance, psychological adaptation, teaching practice

## Abstract

This research establishes and validates an integrative framework to examine the multi-layered responses of higher education to generative AI, focusing on the interplay between institutional governance, pedagogical practices, and student psychological adaptation. Using a student survey sample of 477 respondents across three disciplinary clusters and linking these data to institution- and course-level governance and implementation indicators, we estimate multilevel models and identify response profiles that differentiate adaptation outcomes. The findings indicate that the congruence between governance mandates and classroom execution, bolstered by robust implementation capacity, correlates significantly with enhanced student engagement, self-efficacy, and agency, while concurrently mitigating academic anxiety. Although evidence-based integrity controls facilitate the internalization of ethical norms, they may inadvertently heighten student anxiety in the absence of explicit regulatory frameworks and accessible institutional support. Latent response profiles further reveal that a conditional-aligned, high-support configuration yields the most favorable adaptation pattern, whereas low-clarity and low-support environments correspond to reduced positive adaptation and heightened anxiety. Equity analyses indicate meaningful heterogeneity across institutional tiers and disciplinary contexts, underscoring that effective and fair GenAI governance depends on operational clarity, verification-ready assessment design, and resource-backed support.

## 1 Introduction

The widespread adoption of generative artificial intelligence (GenAI) has begun to alter the fundamental mechanisms of knowledge production and dissemination in higher education. Traditional higher education models have long been built on the assumption of information scarcity and authoritative transmission, with teachers serving as one-way knowledge sources (Wang et al., 2024). Modern technological advancements facilitate the real-time deconstruction and personalized synthesis of knowledge, thereby redirecting pedagogical focus from basic content delivery toward sophisticated instructional design and rigorous critical evaluation (Lo et al., 2024). This transformation not only enhances teaching efficiency but also deeply redefines the essential functions of universities (Deng et al., 2025). Against this backdrop, the connotation of academic skills is undergoing unprecedented evolution. Students are increasingly required to surpass the mere mastery of established theoretical frameworks, as the contemporary landscape demands the capacity to collaborate effectively with intelligent agents to address complex challenges (Jin et al., 2025). This pervasive integration necessitates a reassessment of talent cultivation objectives within the broader context of digital transformation, addressing the structural shifts in educational models driven by technological advancement (van Niekerk et al., 2025).

While generative AI research has proliferated, existing studies predominantly offer fragmented, single-dimensional observations (Al-Abdullatif and Alsubaie, 2024; Türk et al., 2025). Some emphasize macro-level institutional governance or ethical standards, often overlooking practical pedagogical effectiveness. Conversely, micro-level studies frequently focus on psychological determinants—such as technology readiness or anxiety—without accounting for the dynamic influence of macro-institutional environments and meso-level pedagogical interventions (Bai and Wang, 2025; Gonsalves, 2025). This disconnect makes it difficult for current models to explain why identical institutional constraints trigger vastly different psychological adaptation responses.

It must be recognized that the integration of generative AI into educational institutions represents not merely an upgrade of auxiliary tools, but a systemic transformation of socio-technological systems that triggers significant structural disruptions. This paradigm shift has ignited a series of complex and interconnected challenges. First, the evaluation system faces a legitimacy crisis: as AI can now perform traditional academic tasks with high quality, existing assessment frameworks are undergoing fundamental erosion (Foung et al., 2024). Simultaneously, it has spawned a secondary digital divide in equity, where disparities in resource access may further widen cognitive inequalities among learners in the intelligent era. Additionally, teachers’ professional identities are undergoing profound reshaping, transitioning from traditional knowledge transmitters to complex learning curators. This structural reorganization of labor roles can easily induce professional identity confusion (Tbaishat et al., 2025). These issues are not isolated but interpenetrated, collectively forming a complex governance ecosystem. Researchers must transcend singular technological determinism perspectives and examine the holistic transformation of educational ecosystems from a systemic perspective (Freeman, 2025).

Addressing these systemic challenges, this study posits that the capacity of higher education to withstand technological shocks depends largely on fostering sustainable student psychological adaptation through a healthy governance ecosystem rather than simplistic access controls. Theoretically, this integrative framework is anchored in three intersecting paradigms. First, at the macro level, institutional governance is conceptualized through the lens of policy implementation frameworks, emphasizing how top-down institutional mandates are translated into localized, executable practices. Second, at the meso level, teaching practice is grounded in learning theory and instructional design models, highlighting the necessary shift from traditional content delivery toward interactive, technology-enhanced pedagogical innovations (Tripon, 2025). Finally, at the micro level, student psychological adaptation is understood through the intersection of self-regulated learning and technology acceptance theories. This perspectives frames how learners negotiate motivation, academic integrity, and cognitive load in AI-rich environments. By integrating these perspectives, our framework elucidates how structural governance and instructional design synergistically shape students’ self-regulated adaptation and ethical compliance. To this end, we propose an integrative framework that bridges institutional governance, pedagogical practices, and psychological responses. This framework explores how forward-looking institutional governance can serve as a catalyst, driving substantive innovations in teaching paradigms to ultimately reshape students’ psychological adaptation pathways. This research approach not only focuses on enhancing technological efficacy but also emphasizes the coordinated development of learners’ metacognitive abilities, ethical awareness, and emotional resilience.

## 2 Methods

### 2.1 Study design and framework operationalization

This research utilized a student-centered, cross-sectional survey design to investigate the multifaceted responses of higher education institutions to generative artificial intelligence (AI). The study operationalized a conceptual framework designed to evaluate student psychological adaptation and associated learning behaviors within AI-integrated environments. The theoretical framework emphasizes that AI’s impact on learning contexts is not a singular outcome variable, but rather emerges through multiple mechanisms including learners’ cognitive and emotional responses, maintenance of subjectivity, and internalization of ethical norms. These mechanisms ultimately manifest as observable outcomes such as learning engagement, dependency tendencies, and compliance with usage guidelines. For the purpose of this analysis, student psychological adaptation was operationalized as a sustainable and academically compliant state of technology engagement characterized by perceived controllability. This core analytical focus systematically measures dimensions including norm anxiety and uncertainty, learning self-efficacy with AI, subjectivity and authorship, ethical internalization, cognitive load, dependency tendencies, and learning engagement, ensuring strict correspondence between conceptual definitions and measurement indicators.

**Figure 1:**
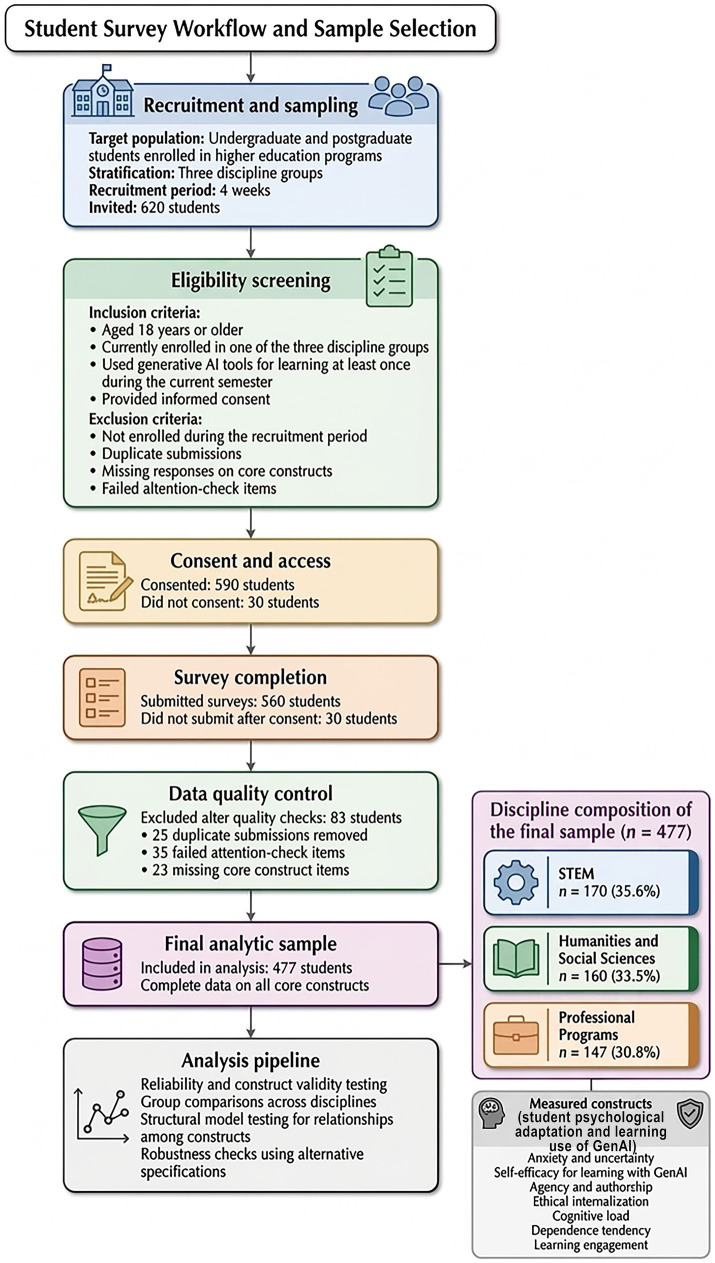
Study workflow and inclusion and exclusion criteria across governance documents, course materials, and student data.

In terms of research implementation, this study employed a stratified sampling strategy to cover three major disciplinary categories: STEM, humanities and social sciences, and specialized disciplines, thereby enhancing the representativeness of the sample structure and cross-disciplinary comparability. The research process strictly adhered to standardized procedures for sample screening and data quality control, encompassing critical stages such as recruitment, informed consent, questionnaire completion, and quality screening. Clear inclusion and exclusion criteria were established to ensure data validity and interpretability. The study subjects were students enrolled in higher education who had actually used generative AI tools during the current semester. Samples with duplicate submissions, missing core constructs, or failing attention checks were excluded to ensure the final sample met the measurement stability requirements for structural modeling and group comparison. The aforementioned research procedures and sample screening logic are fully illustrated in Figure 1, which also summarizes the inclusion/exclusion criteria and sample flow quantities at each stage, providing methodological basis for the transparency and reproducibility of subsequent statistical analyses.

### 2.2 Data sources and sampling strategy

This study utilized student-administered structured questionnaires to assess the application of generative AI in higher education learning contexts and its psychological adaptation. The questionnaire covered key dimensions including AI usage frequency and task types, compliance practices and disclosure behaviors, as well as core psychological adaptation constructs. To ensure data interpretability and statistical stability for structural modeling, a standardized online distribution and collection process was implemented. All participants provided informed consent prior to participation and completed all measurement items and required background variables using the same questionnaire version. The final dataset retained only valid samples meeting inclusion criteria and passing quality control, ensuring the data accurately reflected real-world learning usage patterns and corresponding psychological adaptation characteristics.

A stratified sampling strategy with integrated quota controls was implemented to ensure sample representativeness and facilitate rigorous cross-disciplinary comparisons. The study categorizes disciplinary backgrounds into three major groups: STEM, humanities and social sciences, and specialized disciplines. During the recruitment phase, synchronized enrollment and proportional control are implemented across all three categories to mitigate estimation bias caused by excessive concentration in any single discipline.

Participants were recruited from various higher education programs, encompassing both undergraduate and postgraduate cohorts, with the eligibility criterion requiring at least one instance of GenAI application for academic tasks during the current semester. This approach maintained strong alignment between participants and research objectives. Following a predefined sampling structure, the study ultimately included 477 valid participants: 170 in STEM fields, 160 in humanities and social sciences, and 147 in specialized disciplines. The balanced distribution across these three categories enabled effective comparison of intergroup differences and heterogeneity testing.

Inclusion criteria required participants to be at least 18 years old, enrolled during the recruitment period, belong to one of the three specified academic disciplines, have engaged in generative AI-assisted learning during the current semester, and have completed both informed consent and core questionnaire items. Exclusion criteria included non-enrolled individuals, samples failing disciplinary stratification requirements, duplicate submissions, missing core constructs, and samples failing attention checks. During implementation, questionnaires underwent initial consistency and completeness checks, followed by duplicate submission identification and attention check screening. Core variable data were then filtered to ensure complete observations across key constructs, meeting technical requirements for reliability, validity testing, structural model estimation, and robustness analysis.

### 2.3 Governance coding and measures

This study defines institutional governance as the formal rules and implementation frameworks established by universities regarding the application of generative AI in teaching and learning. Key components include policy positions and scope of application, academic integrity boundaries and disclosure requirements, privacy and data restrictions, accountability and transparency mechanisms, faculty discretion boundaries, as well as training and support measures. The governance framework was validated through a systematic text-coding approach, whereby observable clauses from official institutional policies were extracted and standardized into quantitative indicators suitable for multilevel statistical analysis.

The governance coding framework presented in Table 1 specifies labels, operational definitions, observation indicators, and scoring rules for each governance dimension. The coding process prioritized explicit textual evidence over inferential interpretation, employing an intensity scale to capture the progression from foundational principles to concrete, actionable mechanisms. Based on Table 1, three governance indicators were derived, including policy orientation and scope clarity to reflect institutional boundaries, integrity and accountability mechanisms to assess disclosure processes, and implementation capacity to characterize support infrastructure.

**Table 1:** Coding framework for institutional governance of generative AI in higher education.

Code domain	Code label	Operational definition	Observable indicators	Coding scale and rule	Illustrative policy wording (paraphrased)	Prevalence (institutions, %)	Mean intensity (0–3)
Policy posture	Restrictiveness stance	The overall regulatory stance toward GenAI use in learning/teaching	“Allowed with conditions,” “restricted,” “prohibited in assessed work,” approval requirements	0 = not stated; 1 = permissive; 2 = conditional; 3 = restrictive	“GenAI use is permitted only when explicitly authorized by the instructor and documented.”	76.2	2.21
Scope clarity	Defined scope and scenarios	The degree to which the policy specifies contexts (assignments, exams, research writing, coding)	Scenario list; course/assessment distinctions; discipline notes	0 = none; 1 = generic; 2 = partial list; 3 = detailed scenarios	“Separate rules apply to take-home assignments, timed exams, and thesis writing.”	68.9	1.93
Academic integrity	Integrity classification	Whether GenAI misuse is explicitly mapped to integrity categories (plagiarism, unauthorized assistance, fabrication)	Definitions; examples of violations; boundary language	0 = none; 1 = mentions integrity; 2 = defines misuse; 3 = maps to misconduct categories	“Submitting AI-generated text as one’s own constitutes plagiarism and unauthorized assistance.”	83.4	2.46
Accountability	Enforcement and sanctions	Presence of enforcement pathway and sanction logic	Reporting channels; investigation steps; sanction references	0 = none; 1 = vague; 2 = procedural; 3 = procedural + sanctions	“Suspected misuse is handled under the academic misconduct process with documented outcomes.”	59.5	1.72
Transparency	Disclosure requirement	Whether students must disclose GenAI use and how (statement, appendix, prompt log)	Disclosure template; required elements; when disclosure is mandatory	0 = none; 1 = optional; 2 = required in some tasks; 3 = required broadly + template	“Include an AI-use statement detailing tool, purpose, and extent of modification.”	64.3	1.88
Teaching governance	Instructor discretion boundary	How authority is distributed between institution-level rules and instructor-level rules	“Instructor decides” clauses; minimum institutional baseline	0 = not stated; 1 = full discretion; 2 = discretion within baseline; 3 = strict institutional rule	“Instructors set course-specific rules within the institutional minimum integrity requirements.”	71.8	2.03
Assessment governance	Assessment redesign guidance	Whether the institution provides governance guidance for assessment changes under GenAI	Authentic assessment guidance; process evidence; oral defense; in-class checks	0 = none; 1 = general advice; 2 = recommended practices; 3 = detailed toolkits	“Adopt process-based evidence and authenticity checks for high-stakes assessments.”	52.7	1.54
Privacy and data	Privacy boundary and data handling	Rules restricting sensitive data entry and specifying acceptable platforms/accounts	“No PII/PHI”; approved tools; data retention statements	0 = none; 1 = general caution; 2 = explicit restrictions; 3 = restrictions + approved platforms	“Do not input identifiable student data into external GenAI tools; use approved accounts.”	61.9	1.79
Equity	Access and fairness safeguards	Provisions that address unequal access, accommodations, and fairness in evaluation	Campus provisioned access; alternatives; equity language	0 = none; 1 = acknowledgement; 2 = concrete support; 3 = support + fairness rules	“Provide an alternative pathway when GenAI access is unequal; grading criteria remain comparable.”	34.6	1.11
Capacity building	Training and support infrastructure	Institutional commitment to training for staff/students and support channels	Workshops; helpdesk; teaching center resources; FAQ hubs	0 = none; 1 = *ad hoc*; 2 = structured; 3 = structured + role-based modules	“Offer role-specific training for faculty and students with an implementation support hub.”	57.8	1.63
Update governance	Revision cadence and versioning	Whether documents include update dates, review cycles, or version history	“Last updated”; scheduled review; version control	0 = none; 1 = date only; 2 = date + review plan; 3 = versioned policy cycle	“Policy reviewed each semester; version history maintained for transparency.”	46.9	1.36
Communication	Student-facing clarity	Readability and usability for students (plain language, FAQs, examples)	FAQs; examples; short summaries; visual guides	0 = none; 1 = dense text; 2 = mixed; 3 = student-friendly toolkit	“A student FAQ explains what is permitted, what requires disclosure, and common violations.”	49.8	1.42

### 2.4 Implementation capacity coding and measures

This study defines implementation capacity as the foundational capability of higher education institutions to translate generative AI governance requirements into actionable teaching support. Its core components include guideline operability, training and support systems, accessibility of tools and platforms, privacy and risk management guidelines, as well as fairness and compensation mechanisms. Unlike institutional stances, implementation capacity hinges on whether clear processes, templates, and resources are provided to enable teachers and students to conduct compliant usage and learning activities under consistent rules.

The coding and measurement of implementation capabilities followed a text extraction and indicator development approach. The coding materials primarily include teaching center resource packages, FAQs, training pages, LMS workflow guidelines, support channel announcements, and fair support-related instructions published by universities. The coding framework is presented in Table 2, which clearly defines operational definitions, observable indicators, and scoring rules for each dimension. A strength scale is used to characterize the differences from general principles to actionable implementations.

**Table 2:** Coding framework for institutional implementation capacity (guidelines, training, resources, equity support).

Capacity domain	Code label	Operational definition	Observable indicators	Coding scale and rule	Illustrative institutional practice (paraphrased)	Prevalence (institutions, %)	Mean intensity (0–3)
Guidance quality	Task-specific guidance	Degree to which guidance specifies GenAI use by task type (writing, coding, problem sets, exams)	Task matrix; do/do not lists; discipline notes	0 = none; 1 = generic; 2 = task list; 3 = task list + examples	“Separate guidance is provided for essays, coding assignments, and timed assessments, with concrete examples.”	66.7	1.97
Guidance usability	Student-facing templates	Availability of ready-to-use templates that operationalize rules	AI-use statement template; disclosure checklist; citation format	0 = none; 1 = mention only; 2 = template exists; 3 = template + required fields	“Students submit an AI-use statement specifying tool, purpose, and verification steps.”	52.4	1.61
Faculty support	Teaching-center support	Formal support for instructors to redesign learning and assessment	Consultation hours; course redesign clinics; exemplar repositories	0 = none; 1 = ad hoc; 2 = structured; 3 = structured + exemplars	“Teaching center offers a redesign clinic and a repository of assessment examples.”	59.6	1.74
Training coverage	Faculty training provision	Provision of faculty training with clear scope and schedule	Workshop series; modules; attendance tracking	0 = none; 1 = one-off; 2 = series; 3 = series + role-based tracks	“A multi-week training series provides role-based modules for instructors and TAs.”	57.1	1.69
Training coverage	Student training provision	Provision of student-facing training for responsible GenAI use	Orientation modules; micro-credentials; tutorials	0 = none; 1 = brief note; 2 = training offered; 3 = training + completion pathway	“Students complete a short module on verification, disclosure, and academic integrity.”	45.2	1.33
Tool access	Institution-provided GenAI access	Whether the institution provides approved access pathways	Campus accounts; approved vendors; SSO access	0 = none; 1 = informal suggestion; 2 = approved access; 3 = approved access + guidance	“Approved accounts are provided through SSO with documented acceptable-use conditions.”	38.1	1.18
LMS integration	Workflow integration in LMS	Integration of guidance and disclosure into LMS workflows	LMS plug-ins; assignment prompts; disclosure fields	0 = none; 1 = optional links; 2 = LMS guidance; 3 = embedded workflow fields	“LMS assignment pages include a required AI-use disclosure field for specified tasks.”	31	1.06
Support channels	Student help and reporting routes	Availability of support channels for questions and incident reporting	Helpdesk; dedicated email; reporting form; response SLAs	0 = none; 1 = generic help; 2 = dedicated channel; 3 = dedicated channel + response standard	“A dedicated support inbox and reporting form are monitored with published response times.”	54.8	1.52
Equity safeguards	Equity and accommodation provisions	Measures to mitigate access gaps and ensure fair evaluation	Alternative pathways; device/tool provision; grading guidance	0 = none; 1 = acknowledgement; 2 = concrete support; 3 = support + fairness rules	“When access differs, an equivalent non-GenAI pathway is offered under comparable criteria.”	33.3	1.12
Integrity operations	Process-evidence guidance	Guidance that operationalizes integrity via process evidence	Draft logs; reflection notes; prompt records; oral checks	0 = none; 1 = general; 2 = recommended; 3 = recommended + rubrics	“Guidance requires process evidence and includes rubric language for verification and authorship.”	47.6	1.41
Risk management	Hallucination and verification training	Practical guidance for fact-checking and uncertainty handling	Verification checklist; source triangulation; citation rules	0 = none; 1 = caution note; 2 = checklist; 3 = checklist + worked examples	“A verification checklist is provided with worked examples of error detection and correction.”	61.9	1.83
Privacy operations	Data-protection implementation guidance	Operational instructions to prevent sensitive-data leakage	“Do not upload PII”; approved tools list; redaction steps	0 = none; 1 = generic; 2 = explicit steps; 3 = steps + tool governance	“Guidance specifies redaction steps and restricts use to approved platforms for sensitive content.”	58.7	1.68
Communication	Centralized resource hub	Presence of a single, updated hub that consolidates policies and resources	One-stop portal; navigation; cross-links	0 = none; 1 = scattered links; 2 = partial hub; 3 = centralized hub	“A centralized portal consolidates rules, training, templates, and contact routes.”	50	1.49
Continuous improvement	Feedback and update mechanism	Mechanism to collect feedback and revise guidance	Feedback forms; version notes; scheduled reviews	0 = none; 1 = date only; 2 = feedback channel; 3 = feedback + review cycle	“A feedback channel and semester review cycle are documented with version notes.”	42.9	1.27

### 2.5 Teaching practice coding and measures

Teaching practice in this context refers to the organizational strategies and classroom mechanisms through which GenAI is integrated into the curriculum, particularly regarding its incorporation into instructional design, assessment frameworks, and support systems. Unlike institutional governance, pedagogical practice focuses on operationalizing usable boundaries, evidence requirements, and learning objectives within specific curriculum contexts. Key elements encompassed the clarity and consistency of curriculum rules, disclosure mechanisms, restructured evaluation systems, process evidence, verification standards, instructor feedback, and psychological safety.

Student perceptions of instructional implementation were captured via structured survey instruments, providing quantifiable indicators for subsequent comparative modeling and analysis. The specific coding dimensions, operational definitions, and measurement rules are detailed in Table 3, which also breaks down teaching practice into several testable pathways, covering modules such as course governance, evaluation design, integrity safeguards, teacher instructional behaviors, and support conditions.

**Table 3:** Teaching practice pathways for generative AI integration.

Practice domain	Code label	Operational definition	Example survey indicator (student-reported)	Coding/measurement rule	High implementation (%)	Mean (SD)
Course governance	Clear course-level GenAI rules	The course provides explicit, stable rules on when and how GenAI can be used	“This course clearly states what GenAI use is allowed and what is not.”	Likert 1–5; higher = clearer rules	63.1	3.62 (1.03)
Course governance	Consistency across activities	Rules remain consistent across lectures, assignments, and assessments	“GenAI rules are consistent across different tasks in this course.”	Likert 1–5	55.4	3.39 (1.08)
Transparency	Disclosure requirement	Students are required to disclose GenAI use in submitted work	“I am required to disclose GenAI use in my submissions for this course.”	Likert 1–5	46.8	3.12 (1.21)
Transparency	Disclosure template provided	The course provides a structured template or checklist for disclosure	“A template or checklist is provided for reporting GenAI use (tool, purpose, extent).”	Likert 1–5	38.7	2.91 (1.19)
Assessment design	Authentic assessment emphasis	Assessment design reduces answer-copying by emphasizing context, reflection, or personalized outputs	“Assignments require context-specific reasoning or reflection that cannot be solved by copying.”	Likert 1–5	58.9	3.44 (1.06)
Assessment design	Process-based grading	Grading allocates weight to drafts, iteration logs, or reasoning steps	“The course grades learning process evidence (drafts, iterations, reasoning), not only final answers.”	Likert 1–5	42.1	3.01 (1.17)
Integrity safeguards	Process evidence requirement	Students must submit materials that document how work was produced	“I submit drafts, outlines, revision history, or prompt logs as part of assessment.”	Likert 1–5	34.9	2.78 (1.20)
Integrity safeguards	Verification expectations	The course requires fact-checking and source verification for GenAI-assisted outputs	“The course expects verification of GenAI outputs using credible sources.”	Likert 1–5	61.8	3.55 (1.02)
Integrity safeguards	Citation and attribution rules	Explicit rules exist for citing GenAI assistance and external sources	“The course provides specific rules for citing sources and attributing GenAI assistance.”	Likert 1–5	52.6	3.28 (1.11)
Instructor practice	Constructive feedback on GenAI use	Instructors provide feedback on appropriate use, reasoning quality, and originality	“Feedback addresses how I used GenAI and how to improve my reasoning/original contribution.”	Likert 1–5	47.3	3.17 (1.09)
Instructor practice	Skill-building guidance	Teaching includes strategies for prompting, verification, and critical use	“The course teaches practical strategies for responsible GenAI use (prompting, checking, refining).”	Likert 1–5	44.6	3.06 (1.13)
Assessment governance	Restrictions in high-stakes assessment	GenAI use is restricted or prohibited in exams or high-stakes assessments with clear rationale	“GenAI use is restricted in exams/high-stakes tasks, and the rationale is clearly explained.”	Likert 1–5	69.4	3.81 (0.98)
Support conditions	Access and equity support	The course offers alternatives or support when GenAI access differs among students	“The course provides an alternative pathway if GenAI access is unequal.”	Likert 1–5	29.8	2.63 (1.14)
Learning climate	Psychological safety for disclosure	Students feel safe disclosing GenAI use without fear of unfair penalty when compliant	“I feel safe disclosing GenAI use when I follow the course rules.”	Likert 1–5	51.2	3.31 (1.12)

### 2.6 Student psychological adaptation measures

This study defines student psychological adaptation as a stable regulatory state developed through generative AI-assisted learning. It manifests as the ability to manage uncertainty and anxiety while adhering to academic norms, maintain learning agency and authorship, and transform tool usage into an interpretable and verifiable learning process. Based on this definition, the study employs a questionnaire scale to structurally measure key dimensions of psychological adaptation. Core constructs included norm anxiety and uncertainty, generative AI learning self-efficacy, agency and authorship, ethical internalization, cognitive load, dependency tendency, and learning engagement. Each construct is measured through multiple items, with descriptions focusing on learning task contexts and curriculum requirements to ensure alignment with higher education evaluation and integrity contexts. The questionnaire also collects necessary background variables and usage characteristics to control for potential confounding factors and support intergroup comparisons.

### 2.7 Analytical strategy

The analytical strategy of this study follows a sequential path of “measurement quality confirmation—description and between-group differences—structural relationship testing—robustness testing”. We conducted reliability and validity assessments for each construct, examined internal consistency and construct differentiation of the scale, and performed quality checks on item distribution and missing data to ensure the validity of the measurement model. Subsequently, descriptive statistics were used to conduct comparative analyses across three disciplinary groups, identifying differential patterns of exposure to teaching practices and psychological adaptation indicators under different academic contexts. At the structural level, we tested relationships between psychological adaptation dimensions and the association structure between teaching practices and psychological adaptation. Model estimation employed regression frameworks or structural equation modeling frameworks, reporting standardized effect sizes and confidence intervals, with significant testing conducted on key pathways. Robustness testing was performed to validate conclusion stability, including alternative model specifications, introduction of key control variables, and group sensitivity analyses, thereby enhancing the credibility and reproducibility of the findings.

## 3 Results

### 3.1 Reliability and measurement quality

Systematic reliability and validity assessments were conducted for both text coding and survey measures to ensure rigorous measurement quality. For text coding related to institutional governance and implementation capacity, independent coding by two reviewers was performed with consistency evaluation on selected samples. Analysis revealed high inter-coder consistency across dimensions, confirming the stability and operational feasibility of the coding framework. Both Cohen’s *κ* and Krippendorff’s *α* values for governance and implementation dimensions remained within acceptable ranges and were generally high, confirming consistent recognition of key policy provisions by different coders. This includes policy stance clarity, scope definition, integrity clauses, disclosure requirements, privacy boundaries, and support/training mechanisms. These findings establish a reliable foundation for subsequent text-based indicator development. Detailed coding consistency results are presented in Table 4.

**Table 4:** Inter-coder reliability and construct validity of the multi-layer coding and survey measures.

Measure block	Domain / construct	Double-coded units (*n*)	Cohen’s *κ*	Krippendorff’s *α*	Items	Cronbach’s *α*	CR	AVE	HTMT max
Document coding	Governance: restrictiveness stance	31	0.84	0.81					
Document coding	Governance: scope clarity	29	0.79	0.77					
Document coding	Governance: integrity classification	30	0.86	0.83					
Document coding	Governance: enforcement and sanctions	27	0.76	0.74					
Document coding	Governance: disclosure requirement	28	0.82	0.79					
Document coding	Governance: privacy boundary and data handling	26	0.78	0.75					
Document coding	Governance: equity safeguards	24	0.73	0.71					
Document coding	Implementation: task-specific guidance	34	0.81	0.79					
Document coding	Implementation: student-facing templates	28	0.77	0.75					
Document coding	Implementation: training provision	33	0.79	0.77					
Document coding	Implementation: support channels	29	0.83	0.8					
Document coding	Implementation: verification and risk guidance	31	0.8	0.78					
Document coding	Implementation: privacy operations	27	0.76	0.74					
Survey measures	Anxiety and uncertainty				4	0.87	0.9	0.69	0.76
Survey measures	GenAI learning self-efficacy				4	0.89	0.91	0.71	0.74
Survey measures	Agency and authorship				4	0.85	0.88	0.64	0.79
Survey measures	Ethical internalization				3	0.83	0.87	0.69	0.72
Survey measures	Cognitive load				4	0.81	0.86	0.61	0.77
Survey measures	Dependence tendency				3	0.79	0.85	0.66	0.75
Survey measures	Learning engagement				4	0.86	0.89	0.67	0.78
Survey measures	Teaching practice implementation (course-level exposure)				8	0.88	0.9	0.54	0.81

For student questionnaire measurement, this study conducted internal consistency and construct validity testing on constructs related to psychological adaptation and teaching practice. All constructs displayed robust internal consistency, with Cronbach’s *α* and composite reliability (CR) values consistently exceeding standard thresholds. The average variance extracted (AVE) reached or exceeded common thresholds, supporting convergent validity. Meanwhile, the maximum values of HTMT were consistently below the common discriminant validity threshold, indicating clear differentiation among constructs. This approach avoided excessive overlap between psychological dimensions and teaching practice exposure indicators, thereby reducing interference from homologous measurement in structural relationship estimation.

### 3.2 Governance patterns and typology

Based on the systematic coding results of institutional governance texts, this study categorized the governance orientation of generative AI in higher education institutions, with particular focus on whether clear institutional boundaries have been established and how rule-making authority is allocated between institutional and faculty levels. Descriptive analysis revealed that university governance predominantly featured ambiguous articulation, decentralized rule-making, and conditional permissions, suggesting a transitional phase from abstract principles to executable institutionalization.

**Figure 2:**
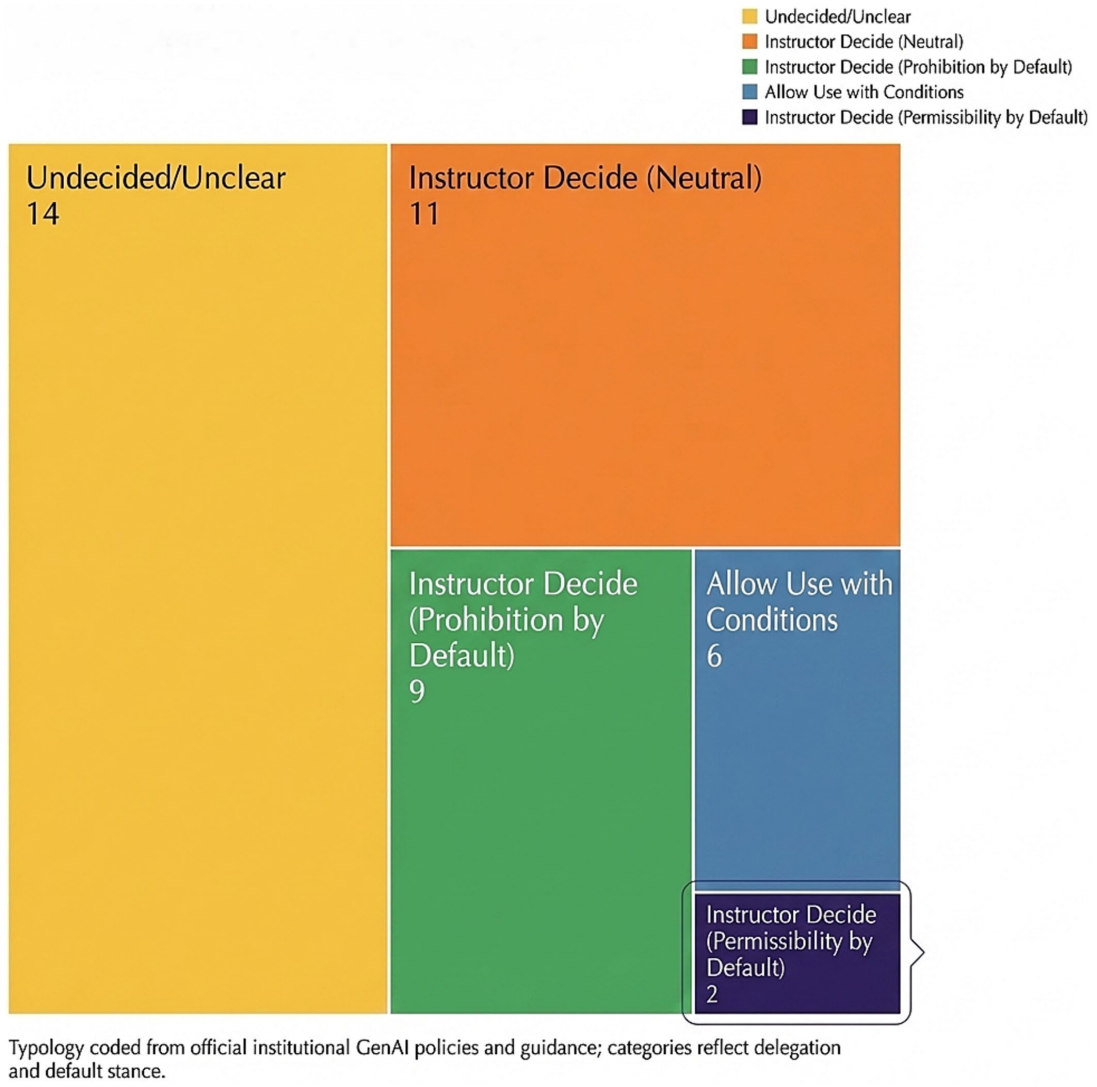
Institutional governance typology of generative AI in higher education.

Regarding category distribution, the plurality of institutions fell within the undefined or ambiguous classification, implying that many entities relied on general guidelines rather than unified, executable rules. The second most common category involves teacher discretion with neutral or prohibited default settings, reflecting universities’ tendency to delegate specific boundaries to course-level decisions. While teachers are expected to establish differentiated regulations based on course objectives and assessment risks, their default stances vary. Some institutions adopt risk control as the baseline, favoring restrictive rules when explicit authorization is lacking, while others opt for relatively neutral authorization methods, emphasizing self-defined permissible scopes. In contrast, conditional allowances represent a relatively low proportion, indicating that few institutions can simultaneously establish clear boundaries, disclosure requirements, and implementation arrangements at the institutional level. The rare occurrence of teacher discretion with default permission highlights that most universities maintain cautious approaches toward academic integrity and assessment validity. The category structure is visually presented in Figure 2.

### 3.3 Governance to pedagogy translation

This study further examined how institutional governance is translated into observable teaching practice signals at the curriculum level, with a focus on the alignment between governance requirements and classroom evaluation mechanisms. Findings indicated that governance mandates did not uniformly permeate instructional processes; rather, they demonstrated characteristics of selective translation. Specifically, alignment was more readily achieved in high-risk, supervised evaluation scenarios, whereas translational efforts remained relatively insufficient in areas such as process-based learning support.

**Figure 3:**
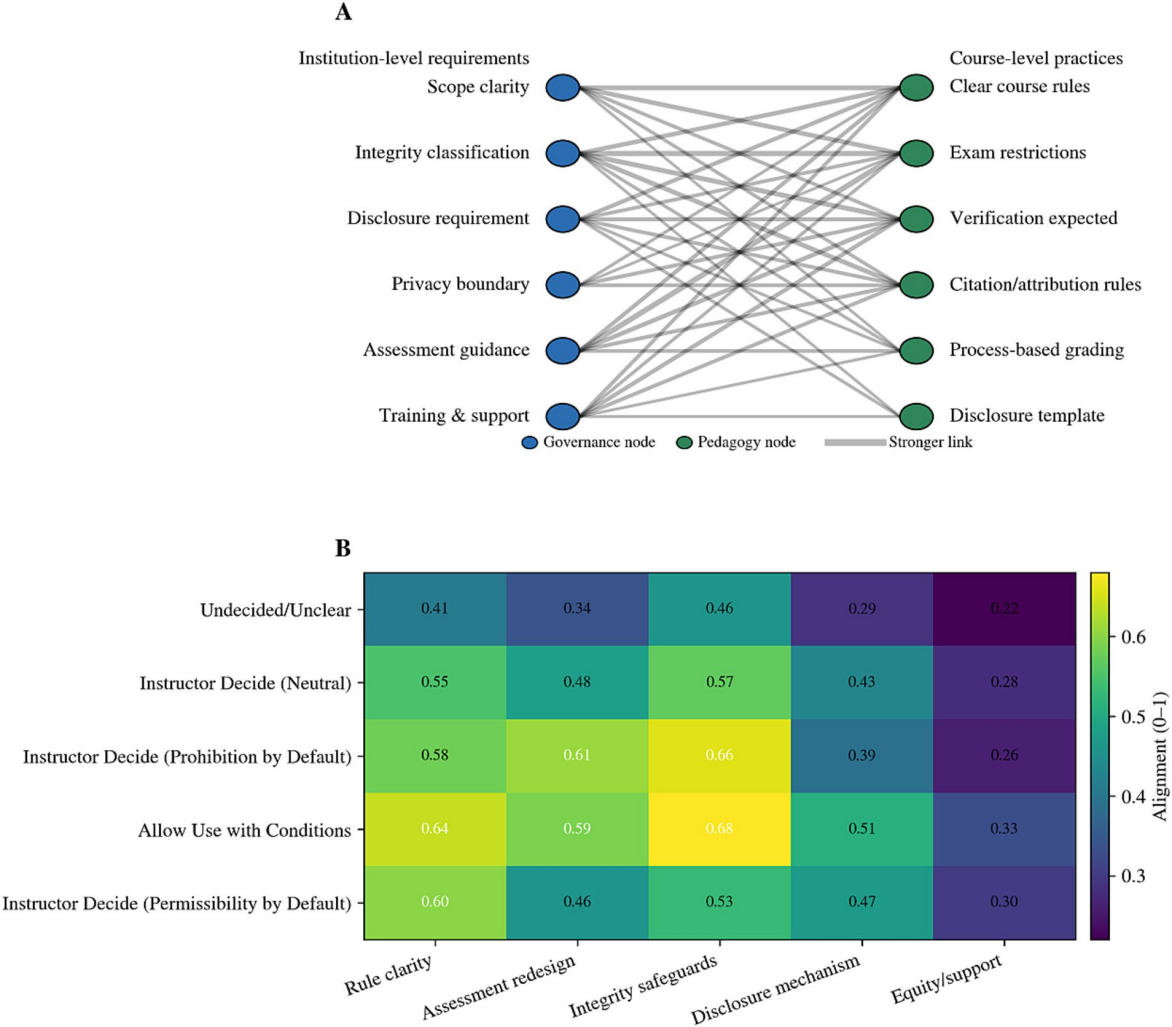
Governance to pedagogy translation map. **(A)** Network diagram showing alignment between institution-level governance requirements (blue nodes) and course-level pedagogical practices (green nodes). Thicker lines indicate stronger alignment. **(B)** Heatmap of alignment scores (0–1) across governance orientations and implementation dimensions. Higher values (yellow) represent stronger alignment.

The translation pathway reveals that institutional frameworks—through their definitions of academic integrity, emphasis on risk assessment, and verification/attribution requirements—establish more stable connections with classroom-level measures like exam restrictions, verification expectations, and citation attribution rules. This pattern reflects a risk-oriented governance strategy, wherein evaluation constraints are prioritized over other implementation domains. In contrast, alignment between disclosure templates, process evidence requirements, and formative assessments remains relatively weak, indicating gaps in translating governance requirements into actionable learning management tools at the course level. Figure 3 illustrates a translation structure characterized by strong evaluation constraints paired with weak process support, where the strength of the policy-to-practice linkage defines the effectiveness of governance implementation.

To quantify the alignment between governance and teaching across universities, this study developed the Governance-Teaching Alignment Index (GPAI) and conducted descriptive statistics and intergroup comparisons at the institutional level. The results indicated that the overall GPAI remained at a moderate level, with systematic differences observed across governance types. Universities with ambiguous governance frameworks demonstrated the lowest alignment levels, suggesting that unclear boundaries undermine consistency and predictability in curriculum implementation. Institutions adopting conditional permission models achieved the highest alignment, indicating that stable implementation signals are more easily established when universities provide clear boundaries, transparent rules, and supporting resources. Universities enforcing default prohibitions with discretionary teacher authority showed above-average alignment, reflecting effective governance implementation through assessment-based restrictions, though deficiencies may persist in disclosure mechanisms and learning support systems. Detailed distribution and grouping results of the GPAI are presented in Table 5.

**Table 5:** Governance–pedagogy alignment index (GPAI): descriptive statistics and cross-institution comparisons.

Grouping variable	Group	Institutions (*n*)	Students (*n*)	GPAI mean	SD	Median	IQR	Min	Max
Overall	All institutions	42	477	0.52	0.11	0.51	0.15	0.29	0.74
Governance typology	Undecided/Unclear	14	163	0.44	0.08	0.45	0.1	0.29	0.58
Governance typology	Instructor Decide (Neutral)	11	131	0.51	0.09	0.5	0.12	0.35	0.67
Governance typology	Instructor Decide (Prohibition by Default)	9	103	0.55	0.1	0.54	0.14	0.37	0.71
Governance typology	Allow Use with Conditions	6	69	0.6	0.09	0.6	0.12	0.45	0.74
Governance typology	Instructor Decide (Permissibility by Default)	2	11	0.57	0.07	0.57	0.1	0.52	0.62
Discipline tier (institution primary focus)	Research-intensive	16	196	0.54	0.11	0.53	0.16	0.31	0.74
Discipline tier (institution primary focus)	Comprehensive	15	172	0.51	0.1	0.51	0.14	0.29	0.69
Discipline tier (institution primary focus)	Teaching-focused	11	109	0.49	0.09	0.49	0.13	0.32	0.66

### 3.4 Course-level governance templates

In the transition from institutional governance to classroom implementation, the syllabus serves as a pivotal institutional vehicle. The curriculum governance template was defined as a rule-based module embedded in syllabi, designed to transform institutional requirements into a comprehensible and verifiable classroom rule system. The core objective of this template was not to standardize all course positions but to provide a replicable framework for minimum viable compliance. This reduces the cognitive burden and compliance pressure caused by fragmented rules across different courses while enhancing the operational feasibility of disclosure, verification, and equitable support mechanisms.

**Figure 4:**
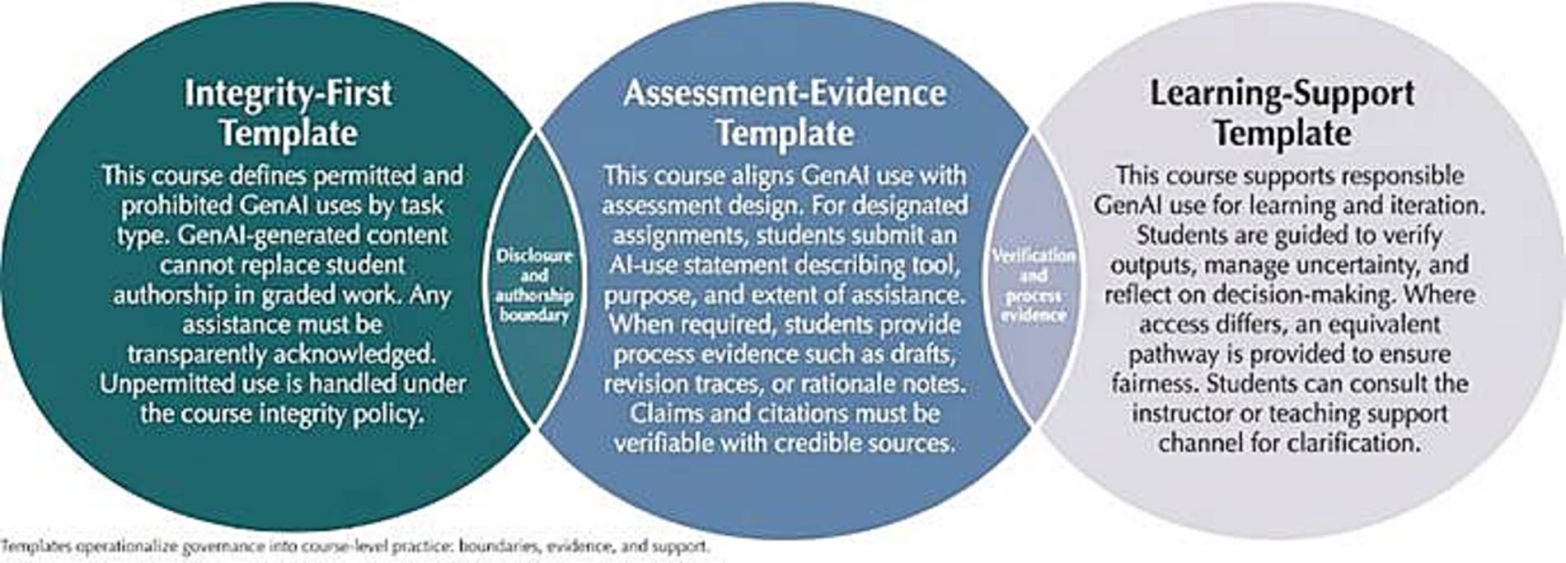
Course-level GenAI syllabus governance templates.

Based on discrepancies between coding results and student feedback, this study identified three representative course governance frameworks: Integrity Boundary, Assessment Evidence, and Learning Support. The Integrity Boundary framework establishes clear boundaries between permissible and prohibited tasks, defining non-disclosure or boundary violations as academic misconduct to create explicit regulatory expectations. The Assessment Evidence framework prioritizes verifiability through AI usage disclosures, process documentation, and traceability requirements, embedding governance into assignment submission and grading systems to reduce reliance on automated detection while enhancing evaluation fairness and transparency. The Learning Support framework encourages students to conduct self-assessment, reflection, and risk management while acknowledging tool accessibility disparities, providing alternative support channels to address resource gaps and prevent algorithmic inequity. These frameworks and their overlapping elements are visually represented in Figure 4, demonstrating how course governance can integrate disclosure mechanisms, usage boundaries, assessment evidence, and student support into modular policy components.

### 3.5 Psychological adaptation structure

This study defined students’ psychological adaptation to generative artificial intelligence as a set of interconnected yet distinguishable psychological and behavioral tendencies, encompassing learning engagement, anxiety, self-efficacy, subjectivity, and ethical internalization. Results suggested that student adaptation was not a binary outcome of acceptance or resistance but manifested as a multidimensional configuration involving both positive resources and perceived risks.

Overall, students demonstrate above-average performance in learning engagement, self-efficacy, and ethical internalization. This indicates that most students have come to view generative AI as a tool for learning support and iterative improvement, while developing a sense of normative awareness and accountability. Conversely, anxiety levels remained moderate, implying that inconsistent rule boundaries and evolving assessment requirements contributed to persistent psychological stress for a subset of students. The subjectivity dimension shows above-average performance, reflecting students’ dual emphasis on efficiency and quality enhancement while also prioritizing control, judgment, and responsibility for outcomes when using the tool.

**Figure 5:**
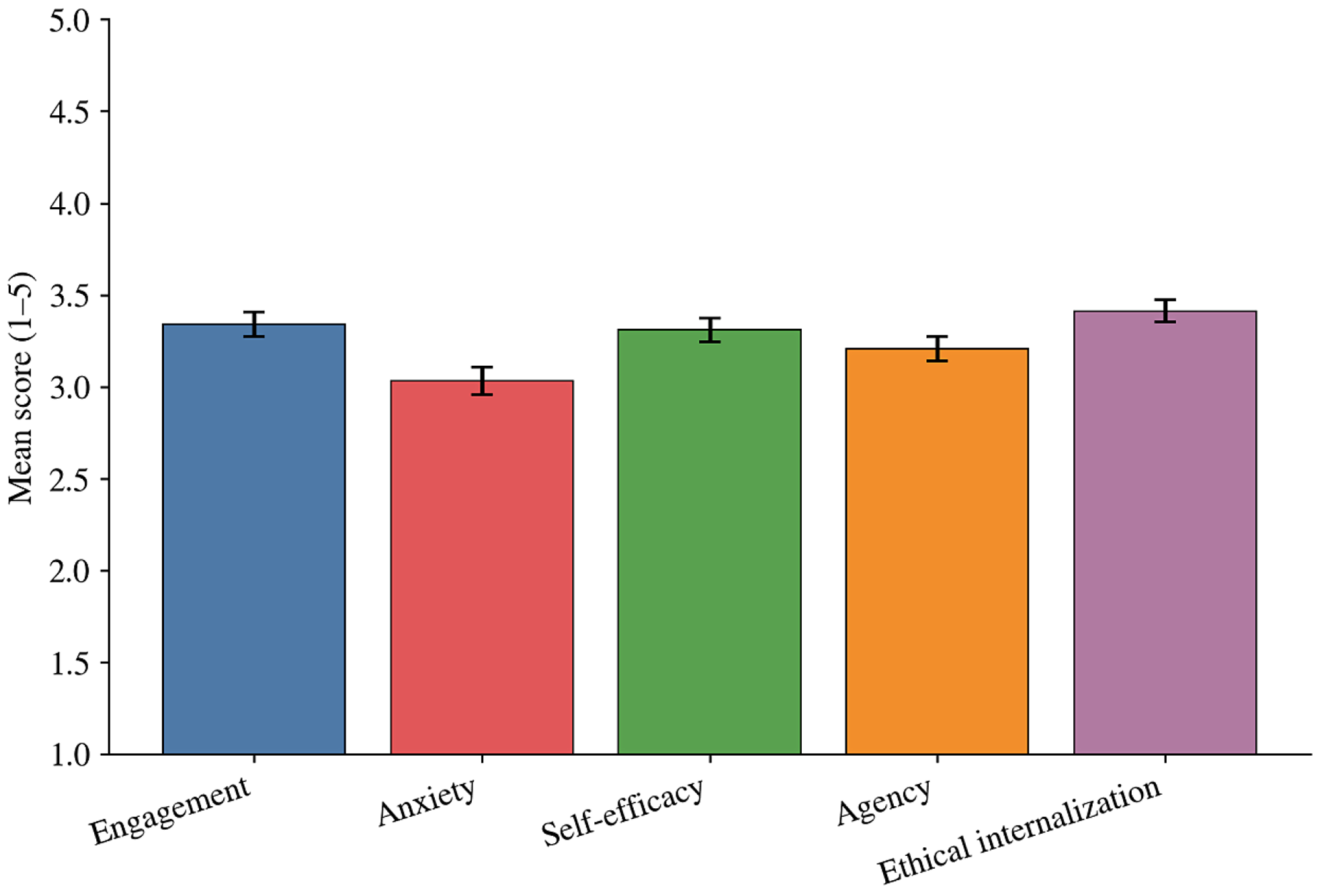
Student psychological adaptation indicators to generative AI.

Figure 5 visually illustrates the distribution of these five indicators. The mean differences across dimensions reveal that students’ adaptation aligns more closely with the structural characteristics of’ efficacy and engagement as primary drivers, with anxiety as a secondary factor.’ Specifically, while positive learning motivation and a sense of competence do not eliminate uncertainty-induced anxiety, they coexist to some extent.

### 3.6 Main model testing

This study employed a two-level multivariate model to examine the impact of institutional governance and teaching practices on students ‘psychological adaptation, while controlling for clustering effects at the university level. Multilevel modeling indicated that institutional governance configurations and implementation capacities functioned as critical determinants of student psychological adaptation, with varying directions of influence across different dimensions.

At the institutional level, universities with well-defined governance frameworks demonstrate more consistent associations between conditional permission and enhanced implementation capabilities, which correlate with higher learning engagement, self-efficacy, and subjectivity, while reducing anxiety. The default prohibition approach may strengthen ethical internalization to some extent, though it is more likely to increase anxiety. The Governance-Teaching Alignment Index (GPAI) emerged as the most consistent predictor across all dimensions, associated with enhanced engagement and efficacy alongside reduced anxiety. This suggests that effective translation of governance into actionable classroom rules would better facilitate students’ high-quality adaptation. These findings are presented in Table 6.

**Table 6:** Multilevel model results linking institutional governance and teaching practice to student psychological adaptation.

Predictors	Engagement *β* (SE) *p*	Anxiety *β* (SE) *p*	Self-efficacy *β* (SE) *p*	Agency *β* (SE) *p*	Ethical internalization *β* (SE) *p*
Level-2 (Institutional)					
Governance typology (ref: Undecided/Unclear)					
Instructor Decide (Neutral)	0.12 (0.05) 0.017	−0.06 (0.05) 0.232	0.10 (0.05) 0.043	0.09 (0.05) 0.071	0.07 (0.05) 0.158
Prohibition by Default	0.08 (0.06) 0.176	0.15 (0.06) 0.011	0.06 (0.06) 0.312	0.04 (0.06) 0.497	0.12 (0.06) 0.049
Allow Use with Conditions	0.19 (0.07) 0.006	−0.13 (0.06) 0.029	0.17 (0.07) 0.012	0.16 (0.07) 0.018	0.14 (0.06) 0.021
Permissibility by Default	0.11 (0.09) 0.214	−0.04 (0.08) 0.618	0.09 (0.09) 0.330	0.10 (0.09) 0.268	0.06 (0.08) 0.446
Implementation capacity index (*z*)	0.16 (0.04) < 0.001	−0.12 (0.04) 0.002	0.14 (0.04) < 0.001	0.13 (0.04) 0.001	0.11 (0.04) 0.006
GPAI (*z*)	0.21 (0.04) < 0.001	−0.18 (0.04) < 0.001	0.19 (0.04) < 0.001	0.17 (0.04) < 0.001	0.15 (0.04) 0.001
Level-1 (Teaching practice perceived by students)					
Clear course rules (*z*)	0.18 (0.03) < 0.001	−0.20 (0.03) < 0.001	0.12 (0.03) < 0.001	0.10 (0.03) 0.001	0.09 (0.03) 0.004
Assessment evidence required (*z*)	0.10 (0.03) 0.002	0.06 (0.03) 0.031	0.08 (0.03) 0.009	0.07 (0.03) 0.016	0.13 (0.03) < 0.001
Learning support availability (*z*)	0.14 (0.03) < 0.001	−0.11 (0.03) < 0.001	0.16 (0.03) < 0.001	0.15 (0.03) < 0.001	0.08 (0.03) 0.010
Controls					
Discipline cluster (ref: STEM)					
Social sciences and humanities	−0.05 (0.04) 0.205	0.06 (0.04) 0.118	−0.02 (0.04) 0.621	−0.03 (0.04) 0.503	0.04 (0.04) 0.296
Health and life sciences	0.02 (0.04) 0.647	0.04 (0.04) 0.334	0.03 (0.04) 0.449	0.01 (0.04) 0.780	0.06 (0.04) 0.129
Prior GenAI use frequency (*z*)	0.17 (0.03) < 0.001	−0.04 (0.03) 0.148	0.15 (0.03) < 0.001	0.13 (0.03) < 0.001	0.05 (0.03) 0.083
Year of study (*z*)	−0.03 (0.02) 0.164	0.05 (0.02) 0.029	−0.02 (0.02) 0.301	−0.01 (0.02) 0.561	0.02 (0.02) 0.318
Random effects and fit					
Institution-level variance (τ00)	0.06	0.07	0.05	0.05	0.04
Residual variance (*σ*^2^)	0.71	0.76	0.68	0.7	0.65
ICC (null model)	0.09	0.1	0.08	0.08	0.06
Institutions / Students	42/477	42/477	42/477	42/477	42/477

### 3.7 Response profiles and outcome differences

This study conducted latent class analysis based on combined indicators of governance and teaching practices. Model fit indices and interpretability criteria supported a four-class solution as the optimal structure. The four response profiles exhibited clear gradients in alignment between governance and teaching, rule clarity, evaluation evidence requirements, and learning support levels, corresponding to significantly different psychological adaptation outcomes (see Table 7).

**Table 7:** Governance and pedagogy response profiles: latent class solutions and associated adaptation outcomes.

(A) Model fit (by number of classes)				
Fit index	*k* = 2	*k* = 3	*k* = 4 (selected)	*k* = 5
AIC	5,621.7	5,520.9	5,478.6	5,472.4
BIC	5,710.3	5,641.2	5,630.7	5,656.2
Entropy	0.78	0.81	0.84	0.80
LMR p	0.003	0.018	0.041	0.267

(B) Class profiles and outcomes (*k* = 4)				
Indicator/outcome	Low-clarity/low-support	Integrity-restrictive/high-evidence	Conditional-aligned/high-support	Permissive-misaligned
Students *n* (%)	149 (31.2)	114 (23.9)	139 (29.1)	75 (15.7)
GPAI (*z*) Mean	−0.58	−0.12	0.64	0.18
Course rule clarity (*z*) Mean	−0.61	−0.18	0.71	−0.06
Assessment evidence required (*z*) Mean	−0.34	0.73	0.28	−0.41
Learning support (*z*) Mean	−0.66	−0.22	0.76	0.02
Engagement Mean ± SD	3.05 ± 0.72	3.28 ± 0.70	3.65 ± 0.69	3.42 ± 0.71
Anxiety Mean ± SD	3.26 ± 0.79	3.32 ± 0.81	2.78 ± 0.75	3.11 ± 0.77
Self-efficacy Mean ± SD	3.06 ± 0.68	3.21 ± 0.66	3.54 ± 0.64	3.33 ± 0.67
Agency Mean ± SD	2.96 ± 0.74	3.07 ± 0.71	3.46 ± 0.68	3.28 ± 0.70
Ethical internalization Mean ± SD	3.18 ± 0.63	3.49 ± 0.58	3.52 ± 0.55	3.21 ± 0.62
Omnibus *p*-value	<0.001	<0.001	<0.001	<0.001

The profile characterized by conditional alignment and high support exhibited the most favorable adaptive outcomes, encompassing elevated engagement and self-efficacy. The integrity-restricted and high-evidence profile showed stronger ethical internalization but relatively higher anxiety levels, suggesting that high constraints and rigorous verification may enhance norm internalization while potentially increasing stress experience. The low-clarity and low-support profile exhibited the lowest performance across most positive adaptation indicators and the highest anxiety, indicating that lack of clear rules and support significantly compromises adaptation quality. The lenient yet mismatched profile fell between these extremes, showing adequate engagement and efficacy, but limited ethical internalization and anxiety reduction due to insufficient rule boundaries and evidence requirements.

### 3.8 Equity and heterogeneity

**Figure 6:**
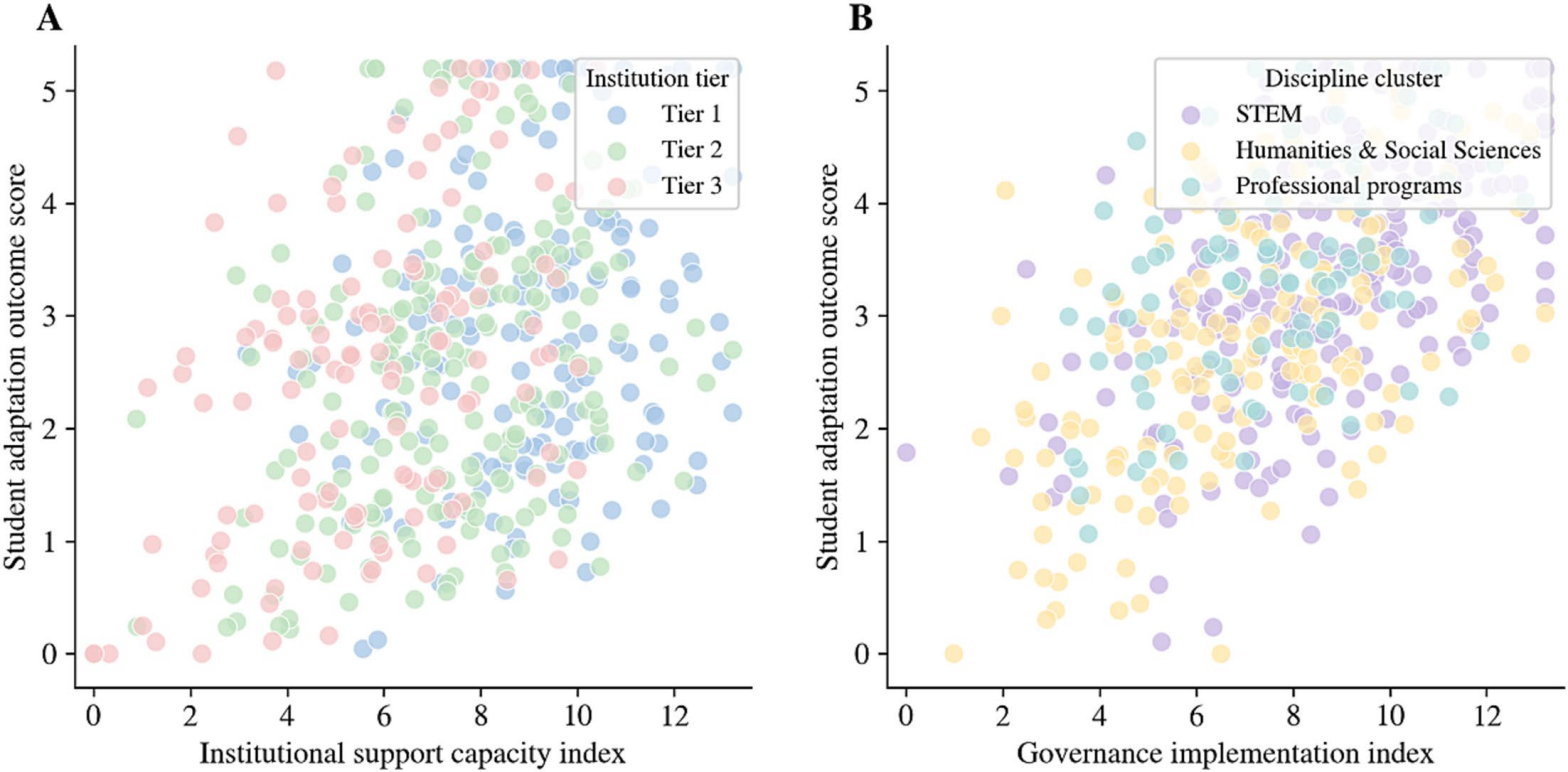
Relationships between institutional governance/implementation indicators and student psychological adaptation outcomes. **(A)** Association between the institutional support capacity index and student adaptation outcome score, with points grouped by institutional tier. **(B)** Association between the governance implementation index and student adaptation outcome score, with points grouped by discipline cluster.

Analysis revealed significant disparities in institutional support and governance implementation across organizational tiers, which aligned with divergent patterns of psychological adaptation. Institutions with stronger support capabilities are more likely to provide accessible training, clear rule explanations, and sustainable teaching resources, thereby achieving higher adaptation levels. Conversely, institutions with insufficient support are more prone to students’ doubts about legitimacy and predictability, which exacerbates anxiety and diminishes engagement and efficacy. Furthermore, disciplinary implementation displayed marked heterogeneity, characterized by structural variations in evaluation methods and perceived integrity risks. This results in varying psychological consequences despite similar governance intensity. The resource accessibility disparities and disciplinary implementation disparities are presented side by side in Figure 6, indicating that equitable governance depends not only on policy statements but also on whether supporting resources and contextualized implementation can meet the actual needs of different institutions and disciplines.

## 4 Discussion

The integration of generative AI into higher education elicited student responses characterized by a complex interplay of value recognition and risk awareness rather than simple acceptance or resistance. Empirical evidence suggests that student psychological adaptation depends less on abstract policy flexibility and more on regulatory clarity, actionable pedagogical practices, verifiable assessment criteria, and accessible support mechanisms (Sousa and Cardoso, 2025). This underscores that effective governance necessitates moving beyond principled declarations to translate institutional signals into coherent, actionable classroom protocols (Wu et al., 2025). By doing so, uncertainties are reduced and predictable learning pathways are established.

From a mechanistic perspective, scenarios characterized by conditional permission and pedagogical alignment correlated strongly with enhanced learning engagement, self-efficacy, and agency, alongside reduced anxiety (Barus et al., 2025; Thaldar et al., 2025). This pattern indicates that clear comprehension of permissible tasks, disclosure standards, and support availability encourages students to perceive generative AI as an instrument for efficiency and quality enhancement, fostering a sense of control and accountability. In contrast, situations with ambiguous rules and insufficient support often correspond to poorer adaptive performance (Azevedo et al., 2025). Regulatory ambiguity increases cognitive load regarding compliance boundaries and fosters inconsistent classroom standards, thereby exacerbating concerns regarding potential violations and evaluation inequity (Dotan et al., 2024). This exacerbates anxiety and suppresses the formation of learning engagement and a sense of competence. Consequently, explicit regulations served as a critical form of psychological support, stabilizing student control over the learning process by mitigating uncertainty (Mollick and Mollick, 2023).

This study further reveals that governance configurations emphasizing academic integrity and evidence requirements demonstrated more pronounced effects in promoting ethical internalization, yet did not necessarily lead to synchronous reductions in anxiety (Zastudil et al., 2023). This suggests that strong normative constraints and low anxiety are not inherently co-extensive. While evidence-based and process-oriented requirements can enhance students’ understanding of authorship and responsibility boundaries, driving them to develop more standardized usage habits, insufficiently clear demonstrations, explanations, or alternative pathways in courses may cause students to perceive evidence requirements as additional burdens, thereby increasing stress to some extent (Bittle and El-Gayar, 2025; Halliday et al., 2025). Effective mitigation involves reframing these requirements as learnable competency frameworks rather than solely as restrictive constraints (Lelescu et al., 2025). For instance, incorporating prompt documentation, version iteration, critical decision rationale, verification steps, and reflective statements into actionable submission structures, along with providing classroom examples and training, helps students clearly understand the relationship between evidence requirements and improved learning quality (Wang et al., 2025). When normative requirements are scaffolded through teaching, students are more likely to perceive compliance as competency development rather than risk avoidance.

Equity and heterogeneity emerged as pivotal findings in this study. Variations in institutional support and resource allocation across organizational tiers significantly influenced student psychological experiences and adaptation outcomes. Environments with abundant resources more readily establish stable training programs, clear procedural guidelines, and sustainable teaching support, thereby enhancing students’ trust in institutional legitimacy and reducing uncertainty-related anxiety (Lee et al., 2024). Conversely, resource-constrained settings may struggle to implement even principled policies due to lack of actionable tools and support channels. Students often develop unease regarding rule comprehension, application boundaries, and evaluation expectations, which subsequently impacts their engagement and sense of efficacy. This demonstrates that equitable governance extends beyond the binary of permission to encompass the provision of equal capacity-building and support conditions.

Disciplinary differences also warrant attention. Our research reveals structural variations in governance implementation and classroom practices across disciplines, resulting in divergent psychological impacts despite similar institutional intensity. Certain disciplines more readily embed usage norms into assessments through procedural and evidence-based approaches, fostering stable student understanding of boundaries. Conversely, others may experience heightened boundary disputes and interpretive disagreements due to diverse task formats and evaluation criteria, intensifying students’ sensitivity and anxiety toward rule consistency. University governance must therefore balance consistency with contextual adaptability, maintaining a foundational framework while permitting the development of interpretable, localized disciplinary rules. Furthermore, teacher training and shared case studies can help mitigate standard drift between courses.

This study acknowledges certain limitations that provide clear avenues for future research. First, our sampling strategy aggregated students into broad disciplinary clusters (e.g., STEM, humanities and social sciences). This necessary grouping may mask inherent heterogeneity within these fields; for instance, the GenAI usage patterns and preferences of computer science students likely differ significantly from those in ecology, just as humanities students may face different assessment paradigms than social science majors. Second, while our sample provides a robust systemic overview, capturing more granular demographic and professional details—such as instructors’ tenure status, years of experience, and baseline comfort with GenAI—would enable deeper interpretation and facilitate strict replication. Future studies should employ more disaggregated sampling strategies and collect comprehensive granular data to unpack these nuanced pedagogical dynamics.

In conclusion, this study advocates an alignment-oriented governance framework. The core principles of this framework center on translating institutional objectives into actionable classroom structures by reducing uncertainty through rule clarity, safeguarding academic integrity via verifiable evaluation chains, and enhancing equity through resource allocation.

## 5 Conclusion

This study established and validated an integrative framework connecting institutional governance, pedagogical practices, and student psychological adaptation. Findings indicated that effective institutional engagement with generative AI depends less on a singular stance of permissiveness or restriction, and more on operational clarity, the alignment of governance with classroom practices, and the accessibility of support infrastructures. Governance configurations characterized by conditional flexibility, high alignment, and robust support were found to foster student engagement, self-efficacy, and agency, while concurrently mitigating anxiety. While evidence-based integrity mechanisms strengthened ethical internalization, they necessitated complementary pedagogical scaffolding to prevent the exacerbation of student stress. Ultimately, the analysis of institutional and disciplinary heterogeneity suggests that equitable governance requires the balanced distribution of capacity-building initiatives and support resources across diverse educational contexts.

## Data Availability

The raw data supporting the conclusions of this article will be made available by the authors, without undue reservation.
